# Understanding Intrinsic Electrochemical Properties of NiCo–Metal–Organic Framework-Derived NiCo_2_O_4_ as a Li-Ion Battery Anode

**DOI:** 10.3390/molecules30030616

**Published:** 2025-01-30

**Authors:** Byoungnam Park, Soomin Kim

**Affiliations:** Department of Materials Science and Engineering, Hongik University, 72-1, Sangsu-dong, Mapo-gu, Seoul 04066, Republic of Korea; ssoomink@g.hongik.ac.kr

**Keywords:** NiCo_2_O_4_, MOF, anode, EPD, additive-free

## Abstract

This study explores the electrochemical properties of additive-free NiCo₂O₄ derived from NiCo–metal–organic frameworks (MOFs) as a high-performance anode material for lithium-ion batteries (LIBs), excluding the effect of additives. NiCo-MOF was synthesized via an ultrasonic-assisted method and deposited on stainless steel foils using alternating current electrophoretic deposition (AC-EPD). The resulting thin films exhibited outstanding cycling stability and rate performance, maintaining a specific capacity of ~1200 mAh/g over 250 cycles at a high current density of 2.35 A/g, with nearly 100% Coulombic efficiency. Differential capacity analysis revealed enhanced redox activity at 0.8 V and 1.7 V during lithiation and delithiation, attributed to the decomposition of NiCo₂O₄ into metallic Ni and Co, followed by their oxidation to Ni^2^⁺ and Co^3^⁺, respectively. The gradual activation of electroactive sites, coupled with improved electrode kinetics and structural adjustments, contributed to the observed capacity increase over cycles. These findings underscore the potential of NiCo₂O₄ as a robust and efficient anode material for next-generation LIBs.

## 1. Introduction

Metal–organic frameworks (MOFs) are inherently porous materials with large specific surface areas [1,2,3]. This feature promotes enhanced interaction with electrolytes and shortens ion transport pathways, significantly benefiting applications like lithium-ion batteries (LIBs) and electrocatalysis [4,5,6,7,8,9]. NiCo-based MOFs are particularly intriguing due to their unique combination of chemical, structural, and electrochemical properties that make them valuable in various applications, including energy storage, electrocatalysis, and solid-state batteries [10,11,12,13]. NiCo MOFs leverage the synergistic properties of Ni and Co, enhancing electronic conductivity and catalytic activity. The combination of these metals often provides improved performance compared to single-metal systems. The structure of NiCo MOFs allows for unsaturated metal sites and tunable pore sizes, providing abundant active sites for redox reactions and catalytic processes. Further, MOF-derived materials, such as NiCo₂O₄ from NiCo MOFs, exhibit high specific capacities and cycling stability in batteries [14,15,16]. These characteristics are attributed to their spinel structures and large surface areas, which enhance lithium-ion intercalation and diffusion [16].

Gunasekaran et al. reported that NiCo MOF-derived porous NiCo₂O₄ nanofile arrays used as an anode exhibited exceptional electrochemical performance, delivering a discharge-specific capacity of 1120 mAh/g at 0.1 C and 210 mAh/g at 2 C after 100 cycles [17]. This impressive performance was attributed to its pseudocapacitive behavior and efficient Li⁺ transport, underscoring its potential as a high-capacity and cycling-stable anode material for rechargeable LIBs. Zhang et al. introduced a synthesis strategy for four distinct NiCo-MOFs with unique structures, achieving high initial discharge capacities ranging from 1770 to 1877.5 mAh/g at 100 mA/g, along with excellent cycling stability [10]. Cheng et al. developed a 3D hierarchical porous Ti₃C₂/NiCo-MOF nanoarchitecture by integrating Ti₃C₂ nanosheets with NiCo-MOF nanoflakes using a vacuum-assisted filtration method [18]. The optimized Ti₃C₂/NiCo-MOF electrode demonstrated outstanding electrochemical properties, including a high reversible capacity of 402 mAh/g at 0.1 A/g after 300 cycles, superior rate capability, and 85.7% capacity retention after 400 cycles. These results highlight the material’s potential for advanced energy storage applications, offering superior performance compared to pristine Ti₃C₂ MXene electrodes.

NiCo MOFs and their derivatives are highly versatile materials for advanced applications, combining the structural advantages of MOF architectures with the unique properties of Ni and Co. While numerous studies have highlighted their potential in energy conversion, storage, and catalysis, the intrinsic electrochemical properties of NiCo-MOF remain relatively underexplored [19,20,21,22].

In this study, additive-free NiCo-MOF films were employed as LIB anodes, fabricated using the alternating current electrophoretic deposition (AC-EPD) method, to explore the intrinsic electrochemical properties of MOF-derived NiCo₂O₄ electrodes without the influence of conductive agents or binders. The use of NiCo-based MOFs as anodes through AC-EPD offers several advantages, combining the unique properties of ultrathin MOFs with the precision and versatility of the EPD technique. The two-dimensional nanosheet structure of NiCo MOFs provides a high surface-to-volume ratio and abundant exposed active sites, facilitating efficient lithium-ion storage and improved reaction kinetics.

The EPD process enables the fabrication of highly uniform and thin films, ensuring greater accessibility of active sites to lithium ions during charge/discharge cycles. NiCo MOFs inherently exhibit enhanced electrical conductivity due to the synergistic interaction between Ni and Co, which is further optimized by EPD, enabling close contact between the material and current collectors.

NiCo-MOF has an intrinsic microporous and mesoporous framework, characteristic of MOF materials, which provides a high surface-to-volume ratio. This structure allows for more exposed active sites, enhancing interaction with ions and reactants. NiCo-MOF is often synthesized in nanosheet or nanoflake forms, further increasing the available surface area for electrochemical reactions. For example, the metal nodes (Ni and Co) combined with organic linkers create a periodic, well-defined porous structure, optimizing surface accessibility.

Further, Ni and Co are transition metals with partially filled d-orbitals, which facilitate electron transfer and enhance the electrical conductivity of the framework. The combination of Ni and Co within the MOF structure provides a synergistic effect, improving electron mobility through the framework. When annealed, NiCo-MOF converts into conductive metal oxides like NiCo₂O₄, further enhancing electrical conductivity while retaining its high surface area. These features make NiCo-MOF an excellent material for energy storage and conversion applications, as it combines efficient ion accessibility with rapid electron transport.

In contrast to conventional DC-EPD, which often results in uneven particle distribution and higher porosity, AC-EPD significantly reduces particle agglomeration by employing a bidirectional electric field, resulting in a smooth, defect-free, and densely packed film [23,24,25].

By leveraging AC-EPD, the uniformity, density, and adhesion of NiCo-MOF coatings on the substrate were significantly improved. The resulting NiCo-MOF films, particularly those derived into NiCo₂O₄, demonstrated excellent specific capacities attributable to their spinel structures and the mixed-valence states of Ni and Co. Furthermore, the intimate contact between the MOF layer and the current collector achieved through EPD minimized interfacial resistance, enabling superior rate capabilities and high-performance cycling stability.

## 2. Results and Discussion

**Figure 1:**
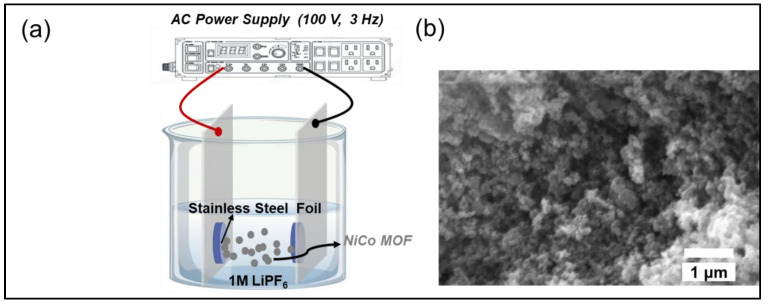
(**a**) Schematic illustration of the AC-EPD setup for fabricating NiCo-MOF films on SS foil using a 100 V, 3 Hz AC power supply. The NiCo-MOF particles are dispersed in 1M LiPF₆ solution during the deposition process. (**b**) SEM image of the NiCo-MOF-derived NiCo₂O₄ thin film deposited on SS foil.

Figure 1 depicts the AC-EPD apparatus, specifically designed for the deposition of battery electrodes, where the setup utilizes an AC voltage operating at a frequency of 4 Hz, ensuring the NiCo MOF powders are evenly distributed onto the SS foil substrate. This configuration is engineered to produce a highly uniform and well-adhered layer, a critical factor for maximizing battery performance.

The alternating nature of the electric field in AC-EPD is instrumental in achieving a homogeneous particle distribution across the substrate. This dynamic electric field prevents the formation of clusters and promotes a smoother coating, as highlighted by the SEM micrograph of the deposited layer in Figure 1. The SEM image depicts the surface morphology of the NiCo₂O₄ film derived from NiCo-MOF, deposited on SS foil via AC-EPD. The individual particles are sub-micrometers in size, consistent with the nanoscale features expected from MOF-derived materials. The visible voids and gaps between the particles suggest a high degree of porosity in the film with a Brunauer–Emmett–Teller surface area of 31 m^2^/g. Smaller particle sizes enhance the electrochemical performance by reducing the ion diffusion path lengths and increasing the number of active sites for lithium-ion storage.

In AC-EPD, the oscillating electric field reverses direction at 4 Hz, influencing the movement of particles towards the substrate in a bidirectional manner. Unlike the unidirectional push in DC-EPD, this alternating motion provides particles multiple opportunities to settle optimally on the surface. This mechanism reduces the likelihood of particles adhering prematurely in suboptimal positions, a common challenge with DC-EPD. Moreover, the bidirectional field prevents the particles from clustering around specific regions of the electrode, leading to a more even and defect-free layer. This improved distribution enhances the structural integrity and bonding strength of the electrode layer, contributing to better electrochemical performance.

**Figure 2:**
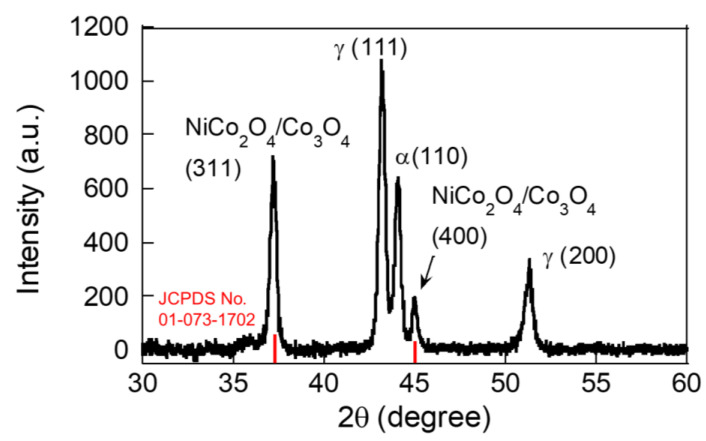
XRD pattern of NiCo-MOF-derived NiCo₂O₄/Co₃O₄ film deposited on SS foil using the AC-EPD method after annealing.

The XRD pattern shown in Figure 2 illustrates the structural characteristics of the NiCo-MOF-derived material deposited on an SS foil via AC-EPD and annealed in a two-step process. Peaks corresponding to NiCo₂O₄ and Co₃O₄ are observed, with their characteristic reflections indexed as (311) and (400) [26]. These peaks indicate the successful formation of spinel-phase materials after the annealing process.

The formation of the Co₃O₄ phase after annealing Ni-Co MOF can occur due to the thermal decomposition and oxidation of cobalt species in the MOF structure during the annealing process. During annealing, the organic ligands in the Ni-Co MOF are thermally decomposed, releasing gases such as CO₂ and H₂O. This exposes the metal centers (Co and Ni) to the environment, enabling phase transformations. At elevated temperatures, cobalt in the MOF can undergo oxidation, forming Co^3^⁺ from Co^2^⁺ through reactions with oxygen from the air during annealing (2Co^2+^ + O_2_ → 2Co^3+^). Co^3^⁺ and Co^2^⁺ coexist in a stable spinel Co₃O₄ structure (containing both Co^2^⁺ and Co^3^⁺), leading to the formation of this phase.

**Figure 3:**
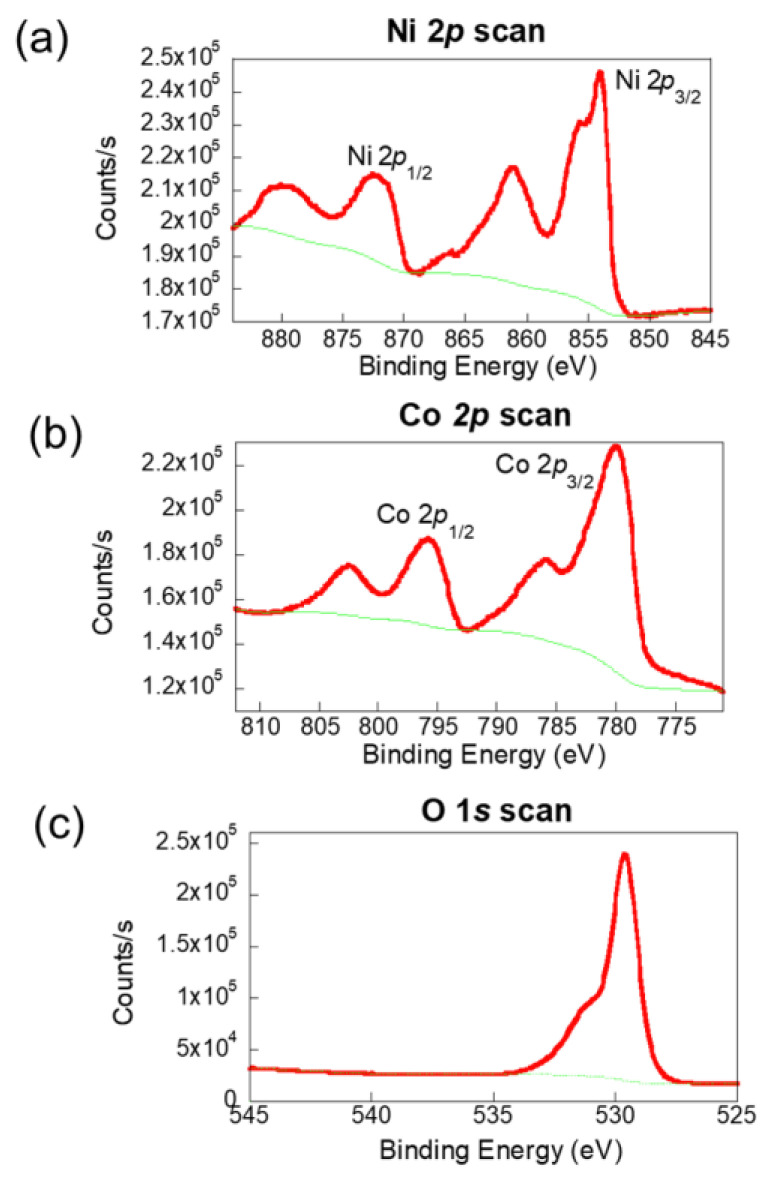
XPS analysis of NiCo-MOF-derived NiCo₂O₄ film deposited via AC-EPD, showing the elemental composition and oxidation states: (**a**) Ni 2p spectrum with peaks for Ni^2^⁺ (Ni 2p₃/₂ and Ni 2p₁/₂) and associated satellite peaks. (**b**) Co 2p spectrum indicating the presence of Co^2^⁺ and Co^3^⁺ with characteristic Co 2p₃/₂ and Co 2p₁/₂ peaks, along with satellite features. (**c**) O 1s spectrum showing peaks for lattice oxygen (metal–oxygen bonds) and surface-adsorbed oxygen species.

**Figure 4:**
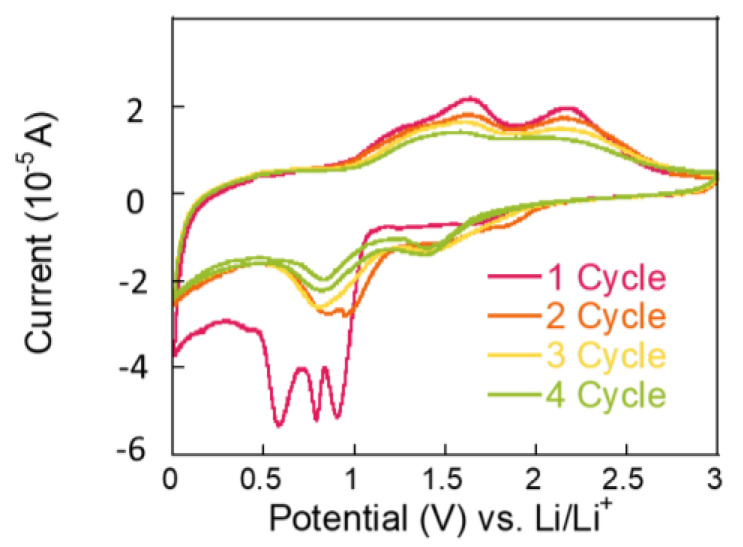
CV curves of the NiCo-MOF-derived NiCo₂O₄ electrode over four cycles at a scan rate of 0.1 mV/s, within a potential range of 0.01–3 V vs. Li/Li⁺.

These phases are known for their excellent electrochemical activity due to mixed-valence states of Ni and Co, which contribute to the redox reactions during lithium-ion storage, which will be discussed in Figure 3 and Figure 4. Peaks labeled γ (111) and α (110) correspond to the austenitic and ferritic SS foil substrate, which may arise from the substrate material or interaction during annealing. The sharpness and intensity of the spinel peaks (e.g., (311) and (400)) suggest good crystallinity of the NiCo₂O₄/Co₃O₄ phases, which is likely due to the high-temperature annealing process. Annealing at 400 °C in air and 600 °C in argon promotes phase formation and removes organic residues from the NiCo-MOF precursor, resulting in crystalline oxide phases. The NiCo₂O₄ spinel structure contributes to high redox activity due to the presence of Ni^2^⁺/Ni^3^⁺ and Co^2^⁺/Co^3^⁺ redox couples. The Co₃O₄ phase enhances the overall electrochemical performance by providing additional active sites for lithium-ion storage. The crystallite size of the NiCo₂O₄ spinel structure was calculated to be 244 nm from the Debye–Scherrer method.

The mixed valence states of Ni and Co in NiCo₂O₄ were investigated by using XPS to analyze the oxidation states of the MOF-derived NiCo₂O₄ film, with the results presented in Figure 3. As shown in Figure 3a, the high-resolution Ni 2p spectrum reveals distinct peaks at 854 and 872 eV, corresponding to the spin-orbit splitting of Ni^3^⁺ 2p₃/₂ and Ni^3^⁺ 2p₁/₂, respectively. Additionally, peaks at 861 and 879 eV are attributed to Ni^2^⁺ 2p₃/₂ and Ni^2^⁺ 2p₁/₂. The Co 2p spectrum, illustrated in Figure 3b, shows two main peaks at 780 and 796 eV, which are indicative of the spin-orbit components Co^3^⁺ 2p₃/₂ and Co^2^⁺ 2p₁/₂. These are accompanied by two satellite peaks, confirming the presence of both Co^3^⁺ and Co^2^⁺ oxidation states. In Figure 3c, the O 1s spectrum highlights a peak at 529 eV, corresponding to the metal–oxygen bonds in the lattice structure. Another peak at 531 eV is associated with surface-adsorbed water molecules. These results collectively validate the mixed-valence states of Ni and Co in NiCo₂O₄ [17].

To investigate the origin of charge storage, redox peaks were analyzed using CV at a scan rate of 0.1 mV s⁻^1^ within a voltage range of 0.01~3 V vs. Li/Li⁺ during the battery charging/discharging process. As shown in Figure 4, the initial scan reveals a reduction peak at approximately 0.6 V, attributed to the reduction in Co₃O₄ to Co during the discharge process. Additionally, sharp reduction (cathodic) peaks between 0.8 and 0.9 V are associated with the decomposition of NiCo₂O₄ into Ni and Co ions, as well as the formation of a solid electrolyte interphase (SEI) layer. In subsequent scans, oxidation of metallic Ni to Ni^2^⁺ and Co to Co^3^⁺ is observed at 1.7 V and 2.2 V, respectively [17,19]. Notably, the charge storage capacity is primarily derived from the conversion reactions, as confirmed by the XRD and CV data. The redox reactions involving NiCo₂O₄ and Co₃O₄ significantly contribute to the overall charge storage capacity, consistent with the proposed Equations (1)–(4).

NiCo_2_O_4_ + 8Li^+^ + 8e^−^ → Ni + 2Co + 4Li_2_O (1)

Ni + Li_2_O ↔ NiO + 2Li^+^ + 2e^−^ (2)

Co + Li_2_O ↔ CoO + 2Li^+^ +2e^−^ (3)

CoO + 1/3Li_2_O ↔ 1/3Co_3_O_4_ + 2/3Li^+^ + 2/3e^−^ (4)

**Figure 5:**
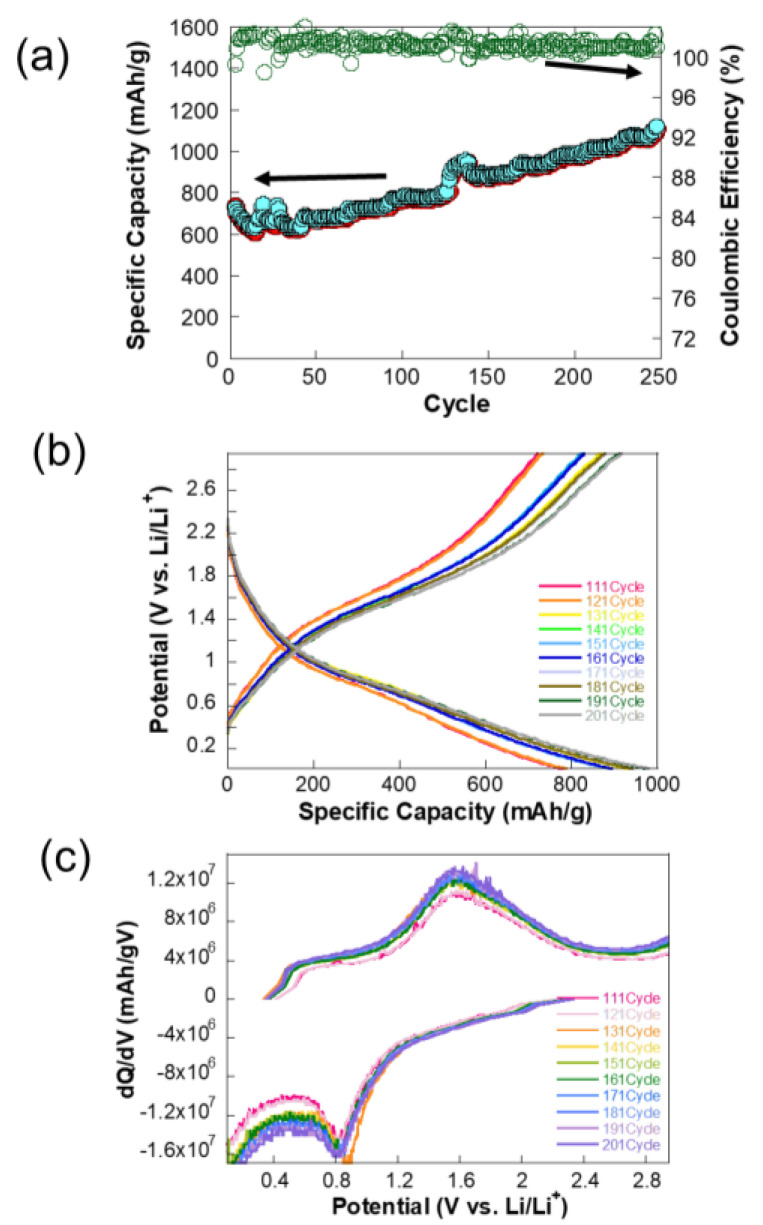
Electrochemical performance of NiCo-MOF-derived NiCo₂O₄ electrodes during cycling: (**a**) Specific capacity and Coulombic efficiency over 250 cycles at a current density of 2.35 A/g. (**b**) Voltage profiles from the 111th to the 201st cycle, highlighting the gradual increase in capacity. (**c**) Differential capacity (dQ/dV) plots, illustrating the redox activity at ~0.8 V (lithiation) and ~1.7 V (delithiation), with increasing peak intensity over cycles.

The plot in Figure 5a illustrates the charge–discharge cycling performance of a NiCo-MOF electrode, tested at a constant specific current of 2.4 A/g. Interestingly, the discharge capacity increases slightly over cycles, which might be due to the activation of additional electroactive sites or gradual improvement in electrode kinetics. After the initial fluctuations, the specific capacity stabilizes around 1100–1200 mAh/g. The Coulombic efficiency is close to 100% throughout the cycling process, indicating minimal irreversible capacity loss and excellent cycling stability. This high efficiency suggests that the charge–discharge process is highly reversible, with minimal degradation of the electrode material.

The initial increase in capacity could be attributed to the formation of a stable SEI or enhanced accessibility of electroactive sites within the NiCo-MOF structure. Over 250 cycles, the capacity remains consistent, indicating the robustness of the NiCo-MOF electrode and its ability to withstand high current densities without significant degradation. The testing current density of 2.4 A/g is relatively high, yet the electrode maintains excellent capacity and stability. This demonstrates the NiCo-MOF electrode’s suitability for high-rate applications, likely due to its high surface area, good conductivity, and structural integrity. The plot highlights the excellent cycling stability, high specific capacity, and outstanding Coulombic efficiency of the NiCo-MOF electrode, even under the demanding conditions of high current density.

The potential profiles in Figure 5b reveal a gradual increase in capacity over cycling, particularly within the voltage range of 0.8 V to 1.7 V. During the discharge process at approximately 0.8 V, this increase is likely attributed to the decomposition of NiCo₂O₄ into metallic Ni and Co ions, where Li⁺ ions react with the metal oxide, leading to its breakdown into metallic components and lithium oxide (Li₂O). Conversely, during the charge process at around 1.7 V, the oxidation of metallic Ni and Co back into Ni^2^⁺ and Co^3^⁺ is observed, corresponding to the delithiation process. This redox activity is characteristic of the spinel NiCo₂O₄ structure. Over successive cycles, the redox reactions in these regions become more pronounced, as evidenced by the increasing capacity. This suggests that repeated cycling activates additional electroactive sites or improves the kinetics of these redox reactions, a phenomenon that will be explored in detail later.

The dQ/dV plot in Figure 5c offers valuable insights into the underlying electrochemical processes. The peak observed around 0.8 V is associated with the lithiation reaction, during which NiCo₂O₄ decomposes into Ni and Co ions. Over successive cycles, the increasing intensity of this peak suggests enhanced lithiation activity, likely due to improved accessibility of electroactive sites or favorable structural adjustments. Similarly, a peak at approximately 1.7 V corresponds to the oxidation of metallic Ni and Co into their higher oxidation states (Ni^2^⁺ and Co^3^⁺) during delithiation. The growing intensity of this peak with continued cycling indicates enhanced reversibility and improved kinetics of the delithiation process.

During early cycles, some active sites within the NiCo₂O₄ structure may not be fully utilized. As cycling progresses, these sites become accessible, contributing to the increased redox activity and overall capacity. The formation and stabilization of an SEI layer may also improve the electrode’s performance by reducing side reactions. Repeated cycling may induce beneficial structural changes in the NiCo₂O₄ material, such as enhanced porosity or breakdown of larger particles, increasing the surface area and enabling more efficient Li-ion transport, which has not been observed in thick electrodes with additives. The enhanced redox peaks in the dQ/dV plot suggest that the lithiation/delithiation kinetics improve over cycles, possibly due to better ionic conductivity or reduced charge-transfer resistance at the electrode–electrolyte interface.

The increase in capacity with cycling is primarily attributed to the improved electrochemical activity at ~0.8 V and ~1.7 V, as evidenced by the potential profile and dQ/dV plot. These regions correspond to the decomposition of NiCo₂O₄ into metallic Ni and Co during lithiation and their re-oxidation during delithiation, as mentioned earlier. Based on the correlation, it is believed that the activation of additional electroactive sites, structural adjustments, and enhanced kinetics over time collectively contribute to this behavior, highlighting the dynamic nature of NiCo₂O₄ as a high-performance electrode material.

The analysis of *b*-values derived from scan rate-dependent CV measurements revealed that surface capacitive contributions dominate the charge storage mechanism at both oxidation (*b* = 0.89) and reduction (*b* = 0.98) potentials [27,28,29]. A *b*-value close to 1.0 indicates that the current is primarily determined by surface-controlled processes, such as pseudocapacitive behavior, rather than diffusion-limited ion intercalation. Indeed, a ratio of 0.37 between diffusive and capacitive charge storage was determined from CV measurements performed at a scan rate of 0.5 mV/s. This highlights the significant role of surface reactions in facilitating rapid charge storage and discharge kinetics in the electrode.

This study presents significant advancements over previous research, underscoring the novelty of our approach [17,30,31]. First, we employed ultrathin NiCo-MOF films, which exhibit exceptional sensitivity to surface electrochemical reactions during charge/discharge processes. This facilitated the development of an advanced platform to probe the electrode/electrolyte interface—an accomplishment unattainable with conventional slurry-cast electrodes due to their bulk nature. Second, we implemented the AC-EPD technique, which has not been previously utilized for NiCo-MOF battery electrodes. This method enabled the fabrication of high-performance electrodes by capitalizing on the pseudocapacitive charge storage mechanism, thereby enhancing energy storage efficiency and rate capability. These innovations highlight the potential of ultrathin NiCo-MOF films and AC-EPD to revolutionize battery electrode design, providing a novel approach to interface analysis and superior electrochemical performance.

## 3. Materials and Methods

### 3.1. Synthesis of NiCo-MOF via Ultrasonic-Assisted Method

NiCo-MOF was synthesized using an ultrasonic-assisted approach. Initially, deionized water (2 mL), ethanol (2 mL), and N,N-dimethylformamide (DMF; 24 mL) were sequentially added to a beaker containing terephthalic acid (PTA, 0.75 mmol). The mixture was stirred continuously until complete dissolution was achieved. Subsequently, nickel (II) chloride hexahydrate (NiCl₂∙6H₂O, 0.375 mmol) and cobalt (II) chloride hexahydrate (CoCl₂∙6H₂O, 0.375 mmol) were simultaneously introduced into the solution and stirred to obtain a homogeneous dispersion. Triethylamine (TEA, 800 µL) was then added dropwise, and the solution was stirred for an additional 30 min.

The reaction mixture underwent ultrasonic treatment for 10 h. During this process, the solution transitioned from a purple color to a cloudy strawberry-milk appearance, indicative of the progression of the reaction. The product was subsequently washed and centrifuged three times with ethanol at 11,000 rpm for 10 min per cycle, with a 10-min ultrasonic treatment applied prior to each centrifugation to ensure uniform dispersion. The purified product was vacuum-dried at 80 °C for 12 h, yielding NiCo-MOF powder. The dried product was further processed in a stainless-steel (SS) mini-ball mill for 10 min to obtain a fine powder suitable for subsequent applications.

### 3.2. Preparation of Electrodes via AC-EPD

The synthesized NiCo-MOF powder was deposited onto SS foil substrates using the AC-EPD technique. For the deposition process, 60 mg of NiCo-MOF powder was dispersed in 30 mL of acetone and sonicated in a bath for 1 h. To ensure a uniform dispersion, tip sonication was performed for an additional 10 min prior to deposition. AC-EPD was conducted at 100 V with a frequency of 3 Hz for 10 min. Following deposition, the electrodes underwent a two-step annealing process, consisting of air annealing at 400 °C for 1 h, followed by argon annealing at 600 °C for 2 h.

### 3.3. Electrochemical and Structural Characterizations

CR2032-type coin cells were assembled within an argon-filled glovebox. The NiCo-MOF electrodes served as the working electrode and were paired with lithium metal as the counter electrode. A liquid electrolyte consisting of ethylene carbonate (EC) and dimethyl carbonate (DMC) in a 1:1 volume ratio, with 1 M LiPF₆ as the lithium salt, was utilized. The electrochemical performance of the assembled cells was assessed through galvanostatic charge–discharge cycling. Each cycle included a charging step, followed by a 10-min rest, a discharging step, and another 10-min rest. The discharge cut-off voltage was set at 0.1 V, while the charge cut-off current was fixed at 50% of the applied current. The current applied to each cell was calculated as the product of the deposited electrode mass and the desired current density. Long-term cycling tests were conducted at a constant current density of 2.35 A/g for the prepared samples. Cyclic voltammetry (CV) was performed at a scan rate of 0.1 mV/s. The NiCo-MOF-derived electrodes were characterized structurally using techniques such as scanning electron microscopy (SEM), X-ray diffraction (XRD), and X-ray photoelectron spectroscopy (XPS).

## 4. Conclusions

The AC-EPD method is a straightforward and scalable technique that can uniformly deposit NiCo MOFs onto various substrates. This makes it a practical approach for fabricating high-performance LIB anodes. The smooth and uniform layers produced by AC-EPD are particularly suitable for integration with solid-state electrolytes, improving overall battery performance. Using NiCo MOFs deposited via AC-EPD as LIB anodes leverages the material’s intrinsic properties—high surface area, tunable porosity, and excellent conductivity—while benefiting from AC-EPD’s precision in creating uniform, efficient, and scalable electrodes. This combination results in enhanced electrochemical performance, structural stability, and potential for high-rate and long-cycle operation, making it a highly promising approach for advanced LIBs.

## Data Availability

Data are contained within the article.

## References

[1] Kitagawa S. (2014). Metal–organic frameworks (MOFs). Chem. Soc. Rev..

[2] Furukawa H., Cordova K.E., O’Keeffe M., Yaghi O.M. (2013). The chemistry and applications of metal-organic frameworks. Science.

[3] Zhou H.-C., Long J.R., Yaghi O.M. (2012). Introduction to metal–organic frameworks. Chem. Rev..

[4] Zhao R., Liang Z., Zou R., Xu Q. (2018). Metal-organic frameworks for batteries. Joule.

[5] KIM Y.-S., CHO H.-I., PARK Y.-E., PARK B.-N. (2020). Fabrication of Fe-Metal Organic Framework-derived Fe_2_O_3_ Nanoparticle Li-ion Battery. Electrochemistry.

[6] Kim Y.J., Park B.-N. (2021). Irreversible capacity loss in additive-free Ni metal organic framework-derived hollow NiO/Ni nanocomposite electrodes as a testbed for energy storage applications. Mater. Lett..

[7] Thakur A.K., Majumder M., Patole S.P., Zaghib K., Reddy M. (2021). Metal–organic framework-based materials: Advances, exploits, and challenges in promoting post Li-ion battery technologies. Mater. Adv..

[8] Sreekanth T., Kiran G., Kim J., Yoo K. (2024). NiCo bimetallic metal-organic framework (NiCo-MOFs) with distinct morphologies for efficient HER activity. Inorg. Chem. Commun..

[9] Shi C., Wang T., Liao X., Qie B., Yang P., Chen M., Wang X., Srinivasan A., Cheng Q., Ye Q. (2019). Accordion-like stretchable Li-ion batteries with high energy density. Energy Storage Mater..

[10] Lin S., Zhang T. (2023). NiCo-MOFs porous nanomaterials prepared by different organic frameworks as anodes for li-ion batteries. J. Energy Storage.

[11] Feng P., Hou W., Bai Z., Bai Y., Sun K., Wang Z. (2023). Ultrathin two-dimensional bimetal NiCo-based MOF nanosheets as ultralight interlayer in lithium-sulfur batteries. Chin. Chem. Lett..

[12] Huang G., Zhang L., Zhang F., Wang L. (2014). Metal–organic framework derived Fe_2_O_3_@NiCo_2_O_4_ porous nanocages as anode materials for Li-ion batteries. Nanoscale.

[13] Wang X., Li Q., Yang N., Yang Y., He F., Chu J., Gong M., Wu B., Zhang R., Xiong S. (2019). Hydrothermal synthesis of NiCo-based bimetal-organic frameworks as electrode materials for supercapacitors. J. Solid State Chem..

[14] Naveen T., Durgalakshmi D., Kishore M.A., Balakumar S., Rajendran A.R. (2025). Rational Design of NiCo_2_O_4_@Carbon Hollow Spheres as a High-Performance Electrode Material for Flexible Supercapacitors. Nanoscale.

[15] Feng X., Huang Y., Li C., Chen X., Zhou S., Gao X., Chen C. (2019). Controllable synthesis of porous NiCo_2_O_4_/NiO/Co_3_O_4_ nanoflowers for asymmetric all-solid-state supercapacitors. Chem. Eng. J..

[16] Chomkhuntod P., Phonsuksawang P., Waehayee A., Ngamchuea K., Iamprasertkun P., Maensiri S., Ruangvittayanon A., Siritanon T. (2024). Effect of solvent-dependent morphology on charge storage mechanism of NiCo_2_O_4_ for aqueous supercapacitors. J. Energy Storage.

[17] Babu S.K., Jayachandran M., Vivek P., Das H.T., Vijayakumar T., Gunasekaran B. (2023). MOF-derived porous NiCo_2_O_4_ nanofile arrays as an efficient anode material for rechargeable Li-ion batteries. J. Alloys Compd..

[18] Liu Y., He Y., Vargun E., Plachy T., Saha P., Cheng Q. (2020). 3D porous Ti_3_C_2_ MXene/NiCo-MOF composites for enhanced lithium storage. Nanomaterials.

[19] Chu K., Li Z., Xu S., Yao G., Xu Y., Niu P., Zheng F. (2020). MOF-derived hollow NiCo_2_O_4_ nanowires as stable Li-ion battery anodes. Dalton Trans..

[20] Xu N., Han Q., Zhu L., Xie L., Xu J., Zhang W., Yang X., Cao X. (2022). Design and synthesis of heterometallic Ni–Co organic frameworks as anode materials for high-performance lithium storage. J. Electrochem. Soc..

[21] Chen J., Jiang J., Liu S., Ren J., Lou Y. (2019). MOF-derived bimetal oxides NiO/NiCo_2_O_4_ with different morphologies as anodes for high-performance lithium-ion battery. Ionics.

[22] Li R., Long Z., Wu C., Dai H., Li W., Bai L., Qiao H., Wang K. (2023). Metal-organic frameworks-derived porous NiCo2O4/carbon composite nanofibers as anodes for Li/Na-ion batteries. J. Alloys Compd..

[23] Lee J., Park B.-N. (2024). Inducing and Understanding Pseudocapacitive Behavior in an Electrophoretically Deposited Lithium Iron Phosphate Li-Metal Battery as an Electrochemical Test Platform. J. Phys. Chem. Lett..

[24] Kollath V.O., Chen Q., Closset R., Luyten J., Traina K., Mullens S., Boccaccini A.R., Cloots R. (2013). AC vs. DC electrophoretic deposition of hydroxyapatite on titanium. J. Eur. Ceram. Soc..

[25] Chávez-Valdez A., Herrmann M., Boccaccini A. (2012). Alternating current electrophoretic deposition (EPD) of TiO_2_ nanoparticles in aqueous suspensions. J. Colloid Interface Sci..

[26] Salunkhe A.D., Pawar P., Pagare P., Kadam A., Katkar P., Torane A. (2023). MOF derived NiCo_2_O_4_ nanosheets for high performance asymmetric supercapacitor. J. Electroanal. Chem..

[27] Guan L., Yu L., Chen G.Z. (2016). Capacitive and non-capacitive faradaic charge storage. Electrochim. Acta.

[28] Park B.-N. (2024). Unraveling Asymmetric Electrochemical Kinetics in Low-Mass-Loading LiNi_1_/_3_Mn_1_/_3_Co_1_/_3_O_2_ (NMC111) Li-Metal All-Solid-State Batteries. Materials.

[29] Kim Y.-J., Park B.-N. (2024). Carrier Depletion-Induced Suppression of Solid Electrolyte Interphase and Conversion Reaction in Zn_1−x_Mg_x_O Nanocrystal Solid Solution Li-Ion Batteries. ACS Appl. Nano Mater..

[30] Li P., Yang H., Wang Q. (2024). Enhanced oxygen evolution reaction and lithium-ion storage performance of MOF-derived NiCo_2_O_4_-NiO-Co@ graphene composites: Effect of carboxylic ligand group. J. Energy Storage.

[31] Xie L., Xu J., Liu M., Han Q., Qiu X., Liu J., Zhu L., Cao X. (2024). Ni-Co MOF-derived rambutan-like NiCo_2_O_4_/NC composite anode materials for high-performance lithium storage. J. Alloys Compd..

